# Relationship Between Criminal and Status Offense Behaviors, Substance Use, and HIV Risk Among Adolescent Girls and Young Women in Cape Town, South Africa

**DOI:** 10.1007/s11469-023-01130-x

**Published:** 2023-08-28

**Authors:** Tara Carney, Bronwyn Myers, Felicia A. Browne, Tracy Kline, Courtney Peasant Bonner, Jacqueline Ndirangu, Wendee M. Wechsberg

**Affiliations:** 1 Mental Health, Alcohol, Substance Use and Tobacco Research Unit, South African Medical Research Council, PO Box 19070, Tygerberg, 7505 South Africa; 2 Division of Addiction Psychiatry, Department of Psychiatry and Mental Health, University of Cape Town, Cape Town, South Africa; 3 Curtin enAble Institute, Faculty of Health Sciences, Curtin University, Perth, WA 6845 Australia; 4 RTI International, 3040 Cornwallis Road, Research Triangle Park, NC 27709 USA; 5 Gillings School of Global Public Health, University of North Carolina, Chapel Hill, NC USA; 6 Department of Psychology, North Carolina State University, NC Raleigh, USA; 7 Department of Psychiatry and Behavioral Sciences, Duke University School of Medicine, NC Durham, USA

**Keywords:** Adolescent girls and young women, Externalizing or challenging behavior, HIV risk, Substance use

## Abstract

This study aimed to examine the relationship between externalizing behaviors, substance use, and sexual risk among adolescent girls and young women (AGYW) in Cape Town, South Africa, who experience social disadvantage characterized by poverty and school dropout. We analyzed baseline data from 500 AGYW in a cluster-randomized trial who had dropped out of school. Multivariate logistic regression models explored associations between self-reported criminal behaviors and other status offenses, heavy episodic drinking, polydrug use, and condomless sex. Engagement in status offenses was associated with heavy episodic drinking (OR = 3.56, 95% CI: 2.05–6.20), while crimes against other people were associated with polydrug use (OR = 1.65, 95% CI: 1.03–2.63). Drug-related illegal behavior was associated with polydrug use (OR = 7.78, 95% CI: 3.53–8.69) and reduced odds of condom use during last sexual episode, after adjusting for drug use (OR = 0.56, 95% CI: 4.00–5.15). As externalizing behaviors are prevalent among this sample of AGYW and associated with greater likelihood of problem substance use and condomless sex, interventions to improve the physical and mental well-being of AGYW should assess for and address engagement in criminal and status offenses.

Physical and mental well-being among adolescent girls and young women (AGYW) provides the foundation for lifelong good health (Chandra-Mouli et al., 2013). In South Africa, which is categorized as a low- and middle-income country (LMIC), high rates of untreated mental health problems have been noted among AGYW in secondary or tertiary education settings (Das-Munshi et al., 2016; Francis & Webster, 2019; Myers et al., 2021a); however, little is known about the mental well-being of out-of-school AGYW. Mental health research among this key population of AGYW in this country has focused principally on internalizing behaviors, such as psychological distress, or symptoms of depression and anxiety (Bonner et al., 2020; Myers et al., 2021b). Research has rarely examined externalizing behaviors (previously referred to as delinquency in the literature). These behaviors include criminal behaviors such as violence, theft, and property damage (Vaughn et al., 2013; White & Renk, 2012), as well as status offenses such as truancy and running away from home (Young et al., 2017).

In addition, most research on criminal and status offenses from high-income countries (HICs) has focused on adolescent boys and young men among whom these behaviors are more prevalent (Hicks et al., 2007; Moffitt, 2018). However, recent evidence suggests this gender gap may be closing. Studies conducted in HICs have found a higher prevalence of these behaviors among AGYW than previously reported (Hicks et al., 2007; Moffitt, 2018; Ogilvie et al., 2020). Notably, research on the prevalence of externalizing behaviors and their impact on the health and well-being of AGYW remains scant in LMICs like South Africa.

South Africa has a long history of economic inequality, with high levels of interpersonal and community violence (Kaminer et al., 2013; Pillay-van Wyk et al., 2016) and school absenteeism and grade repetition (Carney et al., 2013; Pillay-van Wyk et al., 2016), which are more prevalent in communities characterized by high levels of poverty. These are well-known risk factors for engaging in criminal and status offenses and other challenging behaviors such as substance use and sexual risk taking (Abram et al., 2017; Leve et al., 2015; Miller et al., 2016).

In addition, AGYW from historically underserved communities may be at high risk for engaging in these behaviors given their social and educational circumstances. In the Western Cape, AGYW comprise less than half the learners who are still at school by Grade 9, indicating greater risk of dropping out of school early (Department of Basic Education, 2018). This is cause for concern, given evidence from HICs that these criminal and status offenses often co-occur with other behavioral concerns that impact adversely on the health and well-being of AGYW. These behavioral concerns include early pregnancy and sexual risk for HIV (Abram et al., 2017; Leve et al., 2015) with the incidence of HIV for AGYW aged 15–24 being three times higher among AGYW than their male counterparts in this setting (Simbayi et al., 2019), substance use (Abram et al., 2017) such as heavy episodic drinking (Miller et al., 2016; Rocca et al., 2019) and cannabis use (Azad & Ginner Hau, 2018), and mental health issues (Leve et al., 2015).

Limited information is available on the prevalence of criminal and status offenses among AGYW in South Africa who have left school early and how these may be related to other behaviors that increase risk of adverse physical and mental health outcomes. A better understanding of the relationship between criminal and status offenses, substance use, and sexual risk behaviors is needed to inform the design of interventions to reduce South African AGYW’s behavioral risks for HIV. South Africa has long been the global epicenter of HIV, with incidence highest among AGYW (Karim & Baxter, 2019) who are up to five times more likely to be diagnosed with HIV than their male peers (Birdthistle et al., 2019). Despite recent decreases in HIV incidence in the country (Marinda et al., 2020), AGYW remain a key population at risk because of intersecting factors, including gender inequity, sexual risk behavior (condomless sex), and substance use (Carney et al., 2017; Myers et al., 2016).

The impact of these externalizing behaviors may have especially deleterious effects on AGYW whose risk of adverse health outcomes is exacerbated by structural factors, including high levels of poverty, substance use availability, and exposure to violence in their neighborhoods (Donenberg et al., 2020; Watt et al., 2015). This study addresses some of the gap in the literature by exploring associations between criminal and status offenses, substance use, and sexual risk behavior in a sample of AGYW in Cape Town, South Africa, who have left school early. More specifically, the study aims to (1) describe the prevalence of these externalizing behaviors in a cohort study and (2) examine associations between these externalizing behaviors, substance use, and sexual risks for HIV in this vulnerable population.

## Method

Baseline data were drawn from a two-arm cluster-randomized trial, which recruited young women from 24 economically disadvantaged communities (clusters) in Cape Town randomly allocated to an HIV prevention intervention (including HIV testing and counseling) or HIV testing and counseling-only. Clusters were selected from two large geographic areas that varied in the predominant ethnicity. To be included as a cluster, communities had to meet the following eligibility criteria: (1) natural buffers between clusters of at least two streets; (2) cluster consisting of at least 90% of residents with the same ethnicity; and (3) cluster sizes needed to be larger than four streets. Detailed study processes are described elsewhere (Wechsberg et al., 2018).

### Study Sample

From November 2016 to November 2018, 500 eligible young women were recruited by female peer outreach staff. The study sample size was determined by a power analysis based on assumptions based on past studies in South Africa and the USA, as well as assumptions that the primary outcomes are related to risk behaviors (substance use, sexual risk, victimization). For all power analysis, we assumed a two-sided test with *α* = 0.05, a power of 0.8. We also assumed an intracluster correlation coefficient (ICC) estimate of 0.01 to allow for a minimal detectable difference of 0.15 (see Wechsberg et al., 2018). Eligibility criteria included being a woman aged 16 to 19 years old; living in one of the study communities; self-reporting drinking two to three drinks or using any other drug at least once per week over the past 90 days; not attending school, had not attended school for a period of at least 6 months, and had not finished their schooling; and self-reporting condomless sex with a male partner in the past 90 days.

### Procedure

Outreach staff screened young women for eligibility within the study communities. If they were eligible and interested, they were transported to a field site where they were asked to provide written informed consent or assent for study participation. Eligible adolescent girls younger than 18 were accompanied by a trusted woman (mother, guardian, or responsible adult woman from their community) 25 years old and older who provided informed consent and a confidentiality agreement in loco parentis (Bonner et al., 2021). Following the consent process, participants completed the baseline questionnaire that included questions on sociodemographic characteristics, alcohol and other drug use, mental health, and HIV risk behavior. Rapid biological screening for drug use (urinalysis) was conducted.

The questionnaire was administered in English, Afrikaans, or isiXhosa via computer-assisted personal interviewing (CAPI); more sensitive questions were administered through audio computer-assisted self-interviewing (ACASI). The baseline appointment lasted up to two hours and participants were provided an incentive of a grocery voucher worth R150 (~ USD10) for their time. This was in line with South African guidelines for participant reimbursement for health research. Ethics approval was obtained from the relevant ethics committees in South Africa and the USA. There was a study protocol that guided referrals for health and social services and how to manage and support distressed participants at risk of harm.

### Study Measures

#### Engagement in Criminal and Status Offenses

Questions assessed engagement in disruptive, aggressive, and rule-breaking behaviors in the past 6 months, with responses being yes or no. These items were adapted from established self-report delinquency measures (Dembo et al., 2013; Elliott et al., 1985). The original self-reported delinquency measures examined engagement in general theft (including minor or petty theft, vehicle theft, or burglary) and property damage; crimes against persons, such as aggravated assault, fighting, and robbery; drug use sales; and status offenses related to age, such as truancy and running away from home. Categories of behaviors for this study were engagement in the following dichotomous variables: (1) status offenses (underage first line and minor crimes, such as running away, lying in public, and problematic public behavior where others complained about extremely loud or unruly behavior); (2) crimes or injury against persons, such as robbery, physical fights, or physical harm to others; (3) theft and property-related activity, such as trespassing or breaking into property; and (4) drug-related illegal activity, such as drug possession or selling of drugs.

#### Substance Use and Sexual Risk Measures

Recent sexual risk was determined by a dichotomous measure of condom use during last sexual episode. Heavy episodic drinking was defined as four or more alcoholic drinks consumed in a single day during the past 30 days, with response options being yes or no. This measure is based on the National Institute on Alcohol Abuse and Alcoholism’s (NIAAA) guidance on measuring heavy alcohol use in women, which has been widely utilized in previous studies with women in South Africa (Carney et al., 2017; Wechsberg et al., 2017, 2019). Polydrug use, defined as a positive drug screen for two or more illicit drugs (including cannabis; methamphetamine; methaqualone (Mandrax); cocaine; and opiates) was included as an indicator of drug problem severity.

### Data Analysis

Descriptive statistics were conducted on all variables. Tetrachoric correlations revealed that the criminal and status offense items were significantly related. Separate bivariate analyses were conducted to examine baseline associations between categories of these behaviors and sexual risk (sex with a condom at last sex episode), heavy episodic alcohol use, and polydrug use. A complete case analysis was conducted because less than 1% of the data were missing on any of the measures. The sample included in the analyses comprised 498 young women. Chi-square tests of association were performed for categorical data and *t*-tests were conducted for continuous data. Multiple logistic regression analyses were conducted to examine the relationship between categories of criminal offense types and status offenses, and sexual risk and substance use outcomes, while controlling for community cluster with the use of Stata’s survey analysis platform. Sociodemographic variables that were associated with the outcomes at the *p* ≤ 0.10 level were adjusted for in the final regression model. For this model, associations at the *p* ≤ 0.05 level were considered statistically significant. All analyses were conducted in Stata Version 16 (StataCorp, 2019).

## Results

Table 1 presents the sociodemographic characteristics of participants. Their mean age was 17.76 years (standard deviation [SD] = 1.05). Similar proportions of the participants self-identified as Coloured (an official term meaning of mixed ancestry used in SA for population classification) and Black African. More than three-quarters of the AGYW reported that they had a main partner (have a boyfriend: *n* = 384, 77.11%; married: *n* = 6, 1.20%) and just less than half of the participants reported their household ran out of necessities monthly or more often (*n* = 243, 48.80%). Biological testing confirmed that 6.22% (*n* = 31) of the participants were living with HIV and almost all participants reported lifetime alcohol use.

**Table 1:** Sociodemographic characteristics of participants enrolled in Cape Town Young Women’s Health CoOp Study (*N* = 498)

Variables	*N* (%)
Age	*M* = 17.76 (SD = 1.05)
Self-identified ethnicity	
Black African Coloured*	246 (49.40) 252 (50.60)
Unemployed	458 (91.60)
Highest level of education completed	
Grade 8 or lower Grade 9 to 11	150 (30.12) 348 (69.88)
Current relationship status	
Single Main partner (boyfriend/husband)	108 (21.69) 390 (78.31)
Run out of money for necessities monthly or more frequently	243 (48.80)
Living with HIV (biological confirmation)	31 (6.22)
Lifetime alcohol use	496 (99.60)

*Of mixed ancestry

Overall, 80.40% (*n* = 402) of participants reported engaging in some form of criminal or status offense behavior in the previous 6 months, with an average of 2.53 behaviors (SD = 2.40). In terms of the different behavior categories, 73.60% of the participants (95% confidence interval [CI]: 69.9–77.9) reported engagement in status offenses, 47.20% (95% CI: 43.1–52.1) reported crimes or injury against another person, 49.60% (95% CI: 45.5–54.5) reported drug-related illegal behavior, and 22.40% (95% CI: 18.8–26.3) reported involvement in theft and property damage.

Table 2 presents the bivariate associations between sociodemographic factors and the four behavior categories. Significant associations were found between participants’ age and engagement in all four types of criminal behaviors or status offenses, with participants who engaged in status offenses (*t* = 2.17(296); *p* = 0.02), crimes against other people (*t* = 1.71(296), *p* = 0.04), theft and property damage (*t* = 1.73(296), *p* = 0.04), and drug-related sales or possession (*t* = 1.64(296), *p* = 0.05) being significantly younger than participants who had not engaged in these types of behaviors. Leaving school in Grade 9 or earlier was significantly associated with drug-related illegal behaviors only (*χ*^2^(1) = 5.68, *p *= 0.02) in comparison to leaving school after Grade 9. Participants who reported having a current relationship/sexual partner were significantly more likely than those who reported not having a partner to engage in status offenses (*χ*^2^(1) = 6.37, p = 0.01), but not in other types of criminal behaviors. Household hunger was not significantly associated with any of the four behavior categories.

**Table 2:** Description of sociodemographic characteristics by categories of criminal and status offenses (*n* = 498)

Sociodemographic measures	Status offenses (*n* = 368)	*p*-value	Test statistic (*χ*^2^ or t(df))	Crimes against person (*n* = 236)	Test statistic (*χ*^2^ or t(df))	*p*-value	Theft and property damage (*n* = 112)	Test statistic (*χ*^2^ or t(df))	*p*-value	Drug-related illegal behavior (*n* = 248)	Test statistic (*χ*^2^ or t(df))	*p*-value
Age (mean, SD)	17.71 (1.05)	0.02	*t* = 2.17(296)	17.68 (1.06)	*t* = 1.71(296)	0.04	17.61 (1.07)	*t* = 1.73 (296)	0.04	17.69 (1.06)	*t* = 1.64 (296)	0.05
Grade left school *Grade 9 or lower*	*n* = 217 (75.5%)	0.92	*χ*^2^(1) = 0.01	*n* = 144 (61.02%)	*χ*^2^(1) = 0.88	0.35	*n* = 69 (61.61%)	*χ*^2^(1) = 0.46	0.50	*n* = 159 (64.11%)	*χ*^2^(1) = 5.68	0.02
Relationship status *Current partner*	*n* = 278 (75.5%)	0.01	*χ*^2^(1) = 6.37	*n* = 185 (78.39%)	*χ*^2^(1) = 0.02	0.97	*n* = 90 (80.36%)	*χ*^2^(1) = 0.36	0.55	*n* = 192 (77.42%)	*χ*^2^(1) = 0.23	0.63
Hunger in household *Monthly or more frequent*	*n* = 189 (51.36%)	0.61	*χ*^2^(1) = 0.25	*n* = 123 (52.12%)	*χ*^2^(1) = 1.98	0.16	*n* = 61 (54.46%)	*χ*^2^(1) = 1.86	0.17	*n* = 124 (50.0%)	*χ*^2^(1) = 0.29	0.59

Table 3 presents associations between engagement in criminal offense behavior and substance use and sexual risk outcomes. Heavy episodic drinking in the past 30 days was significantly associated with status offenses (*χ*^2^ = 34.11(1), *p* < 0.01), committing crimes against or injuring people (*χ*^2^ = 8.82(1), *p* < 0.01) and theft or damaging property (*χ*^2^ = 4.87(1), *p* = 0.03). Crimes against people (*χ*^2^ = 7.69(1), *p* < 0.01), property damage (*χ*^2^ = 5.03(1), *p* = 0.03), and drug-related illegal behaviors (*χ*^2^ = 84.19(1), *p* < 0.0) were significantly associated with polydrug use. Engaging in drug-related illegal behavior was associated with condom use during last sex (*χ*^2^ = 7.29(1), *p* < 0.01).

**Table 3:** Association between substance use and sexual risk outcomes by types of behavior among young women (bivariate association)

Measures	Heavy episodic drinking (*n* = 415)	Test statistic (*χ*^2^ or t(df))	*p*-value	Polydrug use (*n* = 81)	Test statistic (*χ*^2^ or t(df))	*p*-value	Condom last sex (*n* = 105)	Test statistic (*χ*^2^ or t(df))	*p*-value
Categories of behavior									
Status offenses	328 (79.04%)	34.11 (1)	**< 0.01**	65 (80.26%)	1.96 (1)	0.16	72 (68.57%)	1.96 (1)	0.16
Crimes against person	210 (50.60%)	8.82 (1)	**< 0.01**	52 (54.71%)	10.68 (1)	**0.01**	44 (41.90%)	1.61 (1)	0.21
Theft and property damage	101 (24.34%)	4.87 (1)	**0.03**	28 (34.56%)	8.23 (1)	**0.04**	22 (20.96%)	0.18 (1)	0.97
Drug-related illegal behavior	210 (50.60%)	0.64 (1)	0.42	70 (86.42%)	51.28 (1)	**< 0.01**	40 (38.10%)	7.29 (1)	**< 0.01**

*p*-values in bold to indicate significant differences between associations of the variables listed above and the four categories of offenses

The results from multiple logistic regressions (Table 4) indicate that participants who engaged in status offenses had more than three times greater odds of reporting recent heavy episodic drinking than participants who did not, after adjusting for age (adjusted odds ratio [AOR]: 3.56, 95% CI: 2.05–6.20, *p* < 0.01). The status offenses category was not a significant predictor of the other outcomes (*p*s > 0.05). Engaging in recent crimes against others was significantly associated with polydrug use (AOR: 1.65, 95% CI: 1.03–2.63, *p* = 0.04). Participants who engaged in drug-related illegal activity had significantly greater odds of testing positive for more than one drug compared with participants who did not engage in this illegal activity (AOR: 7.78, 95% CI: 3.53–8.69, *p* < 0.01). Drug-related illegal activity also was significantly associated with decreased odds of condom use at last sex (AOR: 0.56, 95% CI: 0.38–0.82, *p* = 0.01), even after adjusting for drug use.

**Table 4:** Adjusted multiple logistic regression models of criminal and status offenses on substance use and sexual risk outcomes after adjusting for age (controlled for community cluster)

Measures	Heavy episodic drinking			Polydrug use			Condom last sex		
	AOR	95% CI	*p*-value	AOR	95% CI	*p*-value	AOR	95% CI	*p*-value
Categories of behavior									
Status offenses	3.56	2.05–6.20	**< 0.01**	0.91	0.45–1.81	0.78	0.78	0.43–1.40	0.40
Crimes against person	1.42	0.87–2.34	0.16	1.65	1.03–2.63	**0.04**	0.88	0.58–1.29	0.48
Theft and property damage	1.22	0.62–2.38	0.56	1.24	0.60–2.57	0.57	1.20	0.67–2.11	0.54
Drug-related illegal behavior	0.64	0.49–1.30	0.37	7.78	3.53–8.69	**< 0.01**	0.56	0.38–0.82	**< 0.01**

Note. *AOR*, adjusted odds ratio; *CI*, confidence interval

*p*-values in bold to indicate significant differences in odds ratios of the variables listed above and the four categories of offenses

## Discussion

Although associations between criminal and other externalizing behaviors, substance use, and sexual risk have been found among adolescents and young people in high-income countries (HICs) (Hicks et al., 2007; Ogilvie et al., 2020; Popovici et al., 2014; Rocca et al., 2019), limited research exists on how these externalizing behaviors are associated with heavy episodic drinking, polydrug use, and sexual risk among AGYW in LMICs. This study contributes to emerging knowledge on the relationship between types of criminal and status offenses and substance use and sexual risks for HIV within a sample of AGYW who have left school early and live in economically disadvantaged communities in Cape Town, South Africa.

Study findings indicate high rates of criminal and status offenses in our cohort of out-of-school AGYW. Approximately 80% of AGYW in this study reported engagement in at least one of the externalizing behaviors of interest. Even the least prevalent category of criminal and underage challenging behavior (crimes against other people) was reported by just under half of participants. With most mental health research among out-of-school AGYW focusing on the prevalence of internalizing behaviors (Bonner et al., 2020; Myers et al., 2021a) associated with substance use and sexual risks for HIV, our study is among the first to highlight the importance of also addressing externalizing behaviors among out-of-school AGYW in this setting.

More specifically, study findings suggest associations between certain criminal and status offenses and likelihood of polydrug use and heavy episodic drinking, which are both widely recognized as risk factors for HIV among AGYW in South Africa (Mabaso et al., 2018; Wechsberg et al., 2010). In this cohort, even the less severe types of criminal and status offenses were associated with greater likelihood of heavy episodic drinking. Because heavy episodic drinking is characteristic of alcohol use among AGYW in South Africa—with an estimated 40% of young women who drink reporting this pattern of consumption (World Health Organization [WHO], 2018)—and given that this pattern of drinking increases risk of adverse mental and physical health outcomes (Myers et al., 2021b), identifying and reducing risks for this pattern of drinking is critical for the prevention of adverse health outcomes among AGYW who use alcohol. The finding of a significant relationship between condom use at last sex episode and illegal drug-related activities provides additional evidence that AGYW who engage in certain criminal and status offenses may be at increased risk for HIV.

An interesting study finding was that risk for involvement in status offences was higher among participants who reported being in a romantic relationship. While these kinds of offences are generally viewed as less serious than criminal offences, they still reflect behavior that is concerning for their developmental age. It is, however, possible that having one main partner therefore may be a protective factor among AGYW for engaging in criminal activities if this partner is a form of social support. This is agreement with previous studies that found that adolescent girls who reported having significant adults in their lives who cared about them were less likely to report committing these kinds of offenses, although the kind of relationship with this adult was not specified (Hawkins et al., 2009). However, future research should be conducted to explore the role of significant others as protective factors for engagement in risk behavior, including these externalizing behaviors. Findings also point to associations between engaging in violent crimes against other people—such as fighting or hurting others with objects such as knives and guns—and polydrug use. Previous research also has shown that substance use is linked to aggression among adolescent girls (Morojele et al., 2013) and, later on, physical, verbal, and weapon-carrying aggression among adult women (Carney et al., 2017). Addressing involvement criminal and age inappropriate behavior in adolescence may prevent progression to more aggressive and violent behaviors that have more serious consequences for women (Carney et al., 2017).

The associations found between engagement in crime and status offense, substance use, and sexual risk behavior have been documented in justice-involved populations from HICs (Dembo et al., 2017; Sattler, 2017). Our findings confirm that these behaviors generally do not occur in isolation from each other, and that this extends to AGYW who are not yet justice involved. Future research should explore the extent to which these different types of challenging behaviors can be explained by shared risk factors, such as poverty or other forms of early life adversity including exposure to violence and maltreatment. A better understanding of common risk and protective factors is needed to design interventions that target these behaviors (Jessor, 1991, 1992) and prevent AGYW from engaging in more serious offenses and becoming justice involved. More specifically, gender-sensitive and age-appropriate interventions that address gender inequity and unintended pregnancy are needed. Such interventions should target a range of challenging behaviors concurrently. This is particularly important for LMICs that do not have the resources to implement multiple types of preventative interventions, each targeting different challenging behaviors.

Some limitations need to be considered when interpreting these findings. First, a dichotomous, self-report measure of criminal and status offenses was used. We did not assess frequency of engagement in these externalizing behaviors as this was not a primary focus of the parent study. Future studies that focus on externalizing behaviors among AGYW could include more detail on the frequency of engaging in criminal and status offenses and the age at which involvement in these activities began to further investigate this phenomenon (Hale & Viner, 2016). This could assist in establishing degree of involvement and temporality between these and other challenging behaviors. However, a strength of the measures used in the current study was that self-report criminal offenses can identify AGYW who have not yet necessarily engaged with the criminal justice system and therefore may be an opportunity for preventative interventions for AGYW who are often discounted from these types of interventions. Second, while symptoms of mental health difficulties were examined and the results published elsewhere (Bonner et al., 2020; Myers et al., 2021b), we did not assess executive dysfunction or gather information on history of neurodevelopmental disorders, such as attention deficit hyperactivity disorder (ADHD) or other forms of neurodiversity (Retz et al., 2021). This is of particular importance in South Africa which has the highest prevalence of fetal alcohol spectrum disorders (FASD) globally (Olivier et al., 2016). FASD is associated with other neurodevelopment and behavioral challenges such as impulse control and ADHD (Glass et al., 2013) and challenging behaviors during adolescence among boys (Ware et al., 2013) and girls (Kok et al., 2016). Untreated ADHD and other learning difficulties are likely to have been high in this cohort of AGYW who had left school prematurely and used substances. Given the characteristics of this cohort, the high prevalence of criminal or status offenses in this group may not be generalizable to other groups of AGYW in Cape Town or South Africa more broadly. It must also be noted that this study is cross-sectional, so it was not possible to investigate whether criminal or status offenses preceded substance use or condomless sex.

Despite these limitations, study findings highlight high levels of engagement in criminal and status offenses in this cohort of AGYW who use substances and have left school prematurely. Early identification and intervention delivered in a non-stigmatizing manner, when AGYW first begin to experience problems with school attendance, may help prevent progression to more challenging behaviors and keep AGYW retained in school. AGYW who have already disengaged from education may benefit from community-based interventions to reduce involvement in these externalizing behaviors and prevent the progression to criminal behaviors and more adverse consequences. A brief intervention that targets substance use and involvement in externalizing behaviors has been found to be feasible with younger adolescents in this setting (Carney et al., 2020), but is not tailored to the needs of AGYW. This could be adapted to ensure that it addresses the gender-specific needs that AGYW in this setting may have. In summary, early intervention to reduce AGYW’s involvement in externalizing behaviors is critical to prevent progression to more serious offenses that may have violent and legal consequences and impact adversely on AGYW’s physical and mental well-being.

## Data Availability

The data that support the findings of this study are available from Norwegian Social Research (NOVA), but restrictions apply to the availability of these data, which were used under licence for the current study and so are not publicly available. The data are, however, available from the authors upon reasonable request and with the permission of Norwegian Social Research (NOVA).
